# PROTOCOL: Friendly visiting by a volunteer for reducing loneliness and social isolation in older adults

**DOI:** 10.1002/cl2.1084

**Published:** 2020-04-01

**Authors:** Jorien Laermans, Hans Scheers, Philippe Vandekerckhove, Emmy De Buck

**Affiliations:** ^1^ Centre for Evidence‐Based Practice Belgian Red Cross Mechelen Belgium; ^2^ Belgian Red Cross Mechelen Belgium; ^3^ Department of Public Health and Primary Care, Faculty of Medicine KU Leuven Leuven Belgium

## Abstract

Loneliness and social isolation are reaching epidemic proportions in both children and adults, despite the increasing connectedness in our twenty‐first century world. As a growing number of studies reveal their detrimental impact on physical and mental health, identifying and investing in feasible and sustainable interventions to alleviate social isolation and feelings of loneliness is of prime importance. Friendly visiting, a befriending intervention whereby older persons are matched with someone who visits them on a regular basis, seems to be a realistic and sustainable option for providing social support. However, until this day, it remains unclear if friendly visiting by a volunteer is effective at reducing loneliness and social isolation. Therefore, this systematic review aims to answer the following research question: what is the effect of friendly visiting by a volunteer on feelings of loneliness and social isolation (primary outcomes) and wellbeing (i.e. life satisfaction, depressive symptom experiencing and mental health; secondary outcomes) in older adults? The results of this review may provide useful information to policy‐makers that are preparing to take on one the most challenging social issues facing our ageing society.

## 1 BACKGROUND

### 1.1 Description of the condition

The concepts of 'loneliness' and 'social isolation' have been debated and contested extensively, resulting in myriad definitions. In addition, these terms are often used interchangeably, although they are distinct (though related) concepts. Therefore, defining these concepts and highlighting the distinctions between them is of the essence. In this systematic review, loneliness is defined as ‘a subjective, unwelcome feeling of lack or loss of companionship. It happens when we have a mismatch between the quantity and quality of social relationships that we have and those that we want' (the cognitive deficit model of Perlman & Peplau, 1981). It is, therefore, a deeply personal and subjective negative experience. In contrast, social isolation is an objective state, defined in terms of the quantity of social relationships and contacts. It reflects a reduction in social network size and paucity of social contact, which can be triggered by factors such as mobility impairments, unemployment, or deteriorating health (Steptoe, Shankar, Demakakos, & Wardle, 2013). Feeling lonely is therefore different from being socially isolated. In fact, a person may feel lonely even in the presence of other people. Similarly, an individual may live alone without feeling lonely.

Although we live in an increasingly connected world, loneliness and social isolation are reaching epidemic proportions, in both children and adults. The Joint Research Centre of the European Commission reported in a 2018 policy brief that 7% of adults in Europe frequently feel lonely, and 18% (around 75 million people) are socially isolated (i.e. meet socially with friends, relatives or work colleagues at most once a month) (d'Hombres, Schnepf, Barjakovà, & Teixeira Mendonça, 2018). A cross‐country survey of adults in the United States, the United Kingdom and Japan, performed by the Kaiser Family Foundation in partnership with The Economist, revealed that prevalence rates of loneliness or social isolation lie as high as 22% (US), 23% (UK) and 9% (Japan) (DiJulio, Hamel, Muñana, & Brodie, 2018).

These prevalence rates are expected to increase even further during the next couple of decades. Population ageing is one of the key contributors: as people grow older, they are at increased risk of living by themselves and of becoming disabled, which in its turn constitutes a barrier to social interaction. In its 2015 evidence review, age UK stated that 6–10% of older people say they always or often feel lonely, and that nearly half of the people over 65 say that television or pets are their main form of company (Davidson & Rossall, 2015).

An increasing number of studies show that loneliness and social isolation can have a detrimental impact on physical and mental health. For instance, they reportedly have the same harmful effect as smoking 15 cigarettes a day (Holt‐Lunstad, Smith, & Layton, 2010), and put individuals at greater risk of developing clinical dementia (Holwerda et al., 2014). In addition, loneliness has been associated with negative psychological effects such as depressed mood, low levels of life satisfaction and happiness (Prince, Harwood, Blizard, Thomas, & Mann, 1997; Schultz & Moore, 1984). These findings highlight the need for effective interventions to tackle loneliness and social isolation.

A growing range of interventions are being developed to alleviate social isolation and loneliness. These include social facilitation interventions (e.g. friendship clubs, shared interest topic groups), psychological therapies (whereby recognised therapeutic approaches are delivered by trained professionals, e.g. mindfulness, reminiscence therapy), health and social care provision (whereby health and/or social care professionals are involved and participants are enroled in a formal care programme, either in a nursing home or in the community setting), animal interventions (e.g. animal‐assisted therapy, robotic pets), befriending interventions (a form of social facilitation with the aim of formulating new friendships) and leisure/skill development interventions (e.g. gardening programmes, voluntary work, computer training courses) (reviewed by Gardiner, Geldenhuys, & Gott, 2018).

Among the different existing interventions, friendly visiting, a befriending intervention whereby older persons are matched with someone who visits them on a regular basis, seems to be a realistic and sustainable option for providing social support. However, until this day, it remains unclear if friendly visiting by a volunteer is effective at reducing loneliness and social isolation.

### 1.2 Description of the intervention

The intervention of interest for this review is any frequency and any duration of friendly visiting by a volunteer (of any age) to a community‐dwelling or institutionalised older adult. During these visits, the volunteer engages in friendly talking, playing games and/or reminiscing, with the sole purpose of reducing loneliness, social isolation, depressive symptoms, and/or improving life satisfaction and/or mental health in the older adult.

### 1.3 How the intervention might work

The Model of Depression and Loneliness (MODEL) theoretical framework may offer some insight in how friendly visiting might decrease social isolation and loneliness (Cohen‐Mansfield & Parpura‐Gill, 2007). Rooted in a cognitive‐behavioural theory, MODEL describes the influence of environmental resources, health, stressful life events and psychological factors on loneliness and depression in older adults. According to the framework, older adults experience less opportunities to meet people and may face limitations in financial resources, making it harder to create and maintain social contacts. Besides these environmental factors, health issues and difficulties with mobility represent additional barriers to developing meaningful social ties. Stressful life events such as retirement, deaths of friends and family, and relocation can cause people to lose long‐standing social networks, thereby contributing to loneliness. Finally, long‐standing reliance on established contacts, little need to initiate new contacts, and decreased social skills may affect their ability to engage in meaningful social relationships.

The MODEL framework was shown to explain 42% of the variance in loneliness and 47% of the variance in depressed affect among low‐income older adults (Cohen‐Mansfield & Parpura‐Gill, 2007), highlighting the importance of developing interventions targeting the modifiable factors that contribute to loneliness, including new contacts, mobility and financial resources.

Friendly visiting programmes are capable of overcoming most of these barriers, as they allow the older adults to meet and maintain social contact with volunteers, regardless of whether they experience any mobility or financial issues. As loneliness has been associated with depressed mood, low levels of life satisfaction and happiness (Prince, Harwood, Blizard, Thomas, & Mann, 1997; Schultz & Moore, 1984), friendly visiting may exert its beneficial effect on these outcomes through that on loneliness.

### 1.4 Why it is important to do this review

Several existing systematic reviews have looked at the effectiveness of interventions aiming to reduce loneliness or social isolation, but either applied a very broad or a rather narrow scope.

In 2017, a systematic review and meta‐analysis was published investigating the effectiveness of befriending interventions targeting individuals with distressing physical and mental conditions (Siette, Cassidy, & Priebe, 2017). This review included a wide range of befriending interventions (social support delivery through face‐to‐face encounters at home, in support groups, or via telephone contact) in a very diverse population of interest (adults of any age with any type of physical or mental condition).

Similarly, another systematic review on the effectiveness of health promotion interventions that target social isolation and loneliness among older people, used a broad scope for its interventions of interest (Cattan, White, Bond, & Learmouth, 2005). Studies were categorised as ‘group', ‘one‐to‐one', ‘service provision' and ‘community development' interventions. The ‘one‐to‐one' category included a wide range of interventions, including home visits by professionals providing health assessments or services, telephone support‐therapy by social services, friendly telephone calls by peers and social support visits by volunteers.

During the development of the 2015 evidence‐based guideline ‘Older people: independence and mental wellbeing' by the National Institute for Health and Care Excellence (NICE, 2015), another very broadly scoped systematic review was developed to investigate the effectiveness of interventions to improve or protect the mental wellbeing and/or independence of older people in the United Kingdom (McDaid et al., 2015).

Similarly, a recently published integrative review included a wide range of interventions to reduce social isolation and loneliness among older people (Gardiner et al., 2018).

Also in 2018, the What Works Centre for Wellbeing published an overview of 14 systematic reviews of controlled studies published between 2008 and 2018 looking into the effectiveness of interventions aimed to alleviate loneliness (Victor et al., 2018). Again, the included studies investigated an extremely diverse range of interventions, delivered either in the community setting or in care homes and residential facilities.

Despite their broad scopes, none of these existing (overviews of) systematic reviews have allowed to make clear statements on the effectiveness of friendly face‐to‐face visiting by a volunteer to the generalisable older population, that is, older adults that do not suffer from any serious physical or mental illness.

Several other systematic reviews have narrowed the scope of their studied population to adults suffering from chronic non‐cancer pain (Cooper & Wilcock, 2014), only looked at interventions delivered by health or social care professionals (Grant et al., 2014; Montgomery, Mayo‐Wilson, & Dennis, 2008; Sims‐Gould, Tong, Wallis‐Mayer, & Asche, 2017), or did not investigate the effect of friendly visiting (Franck, Molyneux, & Parkinson, 2016; Snowden et al., 2015).

In conclusion, the existing systematic reviews highlight the need for a systematic collection, extraction and analysis of studies looking specifically at the effectiveness of friendly visiting by a volunteer is effective in reducing loneliness and social isolation in older, otherwise healthy, adults. In addition, in their overview of reviews, Victor et al. (2018) highlighted the need for better reporting of numerical data and a focus on effect sizes and precision rather than using *p*‐values as a surrogate for effectiveness, in both future trials and reviews.

Loneliness and social isolation are proving to be among the most challenging social issues to our twenty‐first century ageing society. Given their devastating impact on physical and mental health, policy‐makers should invest in effective interventions to reduce loneliness and social isolation. In January 2018, British Prime Minister Theresa May has set the example, by appointing Tracey Crouch as the country's first Minister for Loneliness. Reviews that study the effects of feasible and sustainable interventions, such as friendly visiting by a volunteer, on loneliness, social isolation and wellbeing, may provide useful information to the minister and other governments and organisations that are preparing to face the challenge.

## 2 OBJECTIVES

By systematically searching for individual studies, this review will answer the following research question:

What is the effect of friendly visiting by a volunteer on feelings of loneliness and social isolation (primary outcomes) and wellbeing (i.e. life satisfaction, depressive symptom experiencing and mental health; secondary outcomes) in older adults?

## 3 METHODS

### 3.1 Criteria for considering studies for this review

#### 3.1.1 Types of studies

Since we will apply quite specific criteria at the level of population and intervention, we will include a broad range of study designs to ensure that the systematic review is as inclusive as possible.

Studies using an experimental design (randomised controlled trials, quasi‐ or non‐randomised controlled trials, controlled before and after studies or controlled interrupted time series) will be included. In addition, we will also include studies using an observational design (cohort studies, case‐control studies, controlled before and after studies, controlled interrupted time series, cross‐sectional studies), as we anticipate that they will provide the majority of the available evidence.

Other study designs such as case series, narrative reviews and non‐original studies such as editorials, book reviews, commentaries and letters to the editor, will be excluded.

In addition, qualitative studies will not be included in this review.

#### 3.1.2 Types of participants

Studies in community‐dwelling and institutionalised older adults (≥60 years of age) will be included. Studies that also include younger adults (<60 years of age) will only be included if: (a) they report the results separately for ≥ 60‐year‐olds, or (b) they specifically define the population as ‘older adults' or ‘elderly' and the average age of the participants is or exceeds the age of 60.

As this review will be conducted to directly inform the friendly visiting programme of the Belgian Red Cross, which specifically aims at tackling loneliness within the general population of older adults, studies focusing exclusively on specific groups, such as widow(er)s or bereaved older adults, caregivers of older adults, hospitalised older adults, community‐dwelling older adults with severe mental or physical health problems (e.g. palliative care patients, clinically depressed older adults), are beyond the scope of this review.

#### 3.1.3 Types of interventions

Interventions for this systematic review will include any frequency and any duration of friendly visiting by a volunteer (of any age) to an older adult (≥60 years of age). These visits should consist of friendly talking, playing games and/or reminiscing, with the sole purpose of reducing loneliness, social isolation, depressive symptoms, and/or improving life satisfaction and/or mental health in the older adult.

Interventions delivered by health or social care professionals will be excluded from the review. As this review aims at investigating the effect of face‐to‐face social interaction with others, interventions delivered via computerised systems or telephone will be excluded as well. In addition, studies concerning screening of older adults, small group meetings, support groups, social networks, extensive courses, computer courses at home and support for the bereaved will be excluded.

Within experimental studies, the effect of friendly visiting will be compared to no friendly visiting. For observational studies, the outcomes (see below) of older adults who received friendly visits will be compared to those of older adults who did not receive friendly visits.

#### 3.1.4 Types of outcome measures

Studies will be included if they have quantitatively measured the effect of friendly visiting on at least one or more of the following primary or secondary outcomes.

#### 3.1.5 Primary outcomes

The primary outcomes for this review are loneliness and social isolation.

Studies that have measured loneliness will be included, regardless of the measurement instrument used. Loneliness measuring instruments include, but are not limited to:

• Validated formal loneliness scales:
  ◦ UCLA 20‐Item Loneliness Scale (Russell, 1996);
  ◦ UCLA 3‐Item Loneliness Scale (Hughes, Waite, Hawkley, & Cacioppo, 2004);
  ◦ De Jong Gierveld 11‐Item Loneliness Scale (De Jong Gierveld & Kamphuis, 1985; De Jong Gierveld & van Tilburg, 1999);
  ◦ De Jong Gierveld 6‐Item Loneliness Scale (De Jong Gierveld & Van Tilburg, 2006);
  ◦ Social and Emotional 37‐Item Loneliness Scale for Adults (SELSA) (DiTommaso & Spinner, 1993);
  ◦ Social and Emotional 15‐Item Loneliness Scale for Adults (SELSA‐S) (DiTommaso, Brannen, & Best, 2004).
• Single‐item questions, such as:
  ◦ How often do you feel lonely? (hardly ever or never, some of the time, often);
  ◦ During the past week, have you felt lonely? (rarely or none of the time (e.g. less than 1 day), some or a little of the time (e.g. 1–2 days), occasionally or a moderate amount of time (e.g. 3–4 days), all of the time (e.g. 5–7 days).

Studies that have measured social isolation will be included, as long as the measuring instrument used objectively quantifies social isolation (i.e., by measuring the frequency of social contact and/or the size of the respondent's social network). Objective social isolation measuring instruments include, but are not limited to:

• Validated scales:
  ◦ Lubben Social Network 10‐Item Scale (Lubben, 1988);
  ◦ Lubben Social Network 6‐Item Scale (Lubben et al., 2006).
• Single‐item questions, such as:
  ◦ How often do you meet socially with friends, relatives or work colleagues?
  ◦ How often do you have contact with non‐cohabitant others?

Studies using instruments that measure social support in a subjective way (i.e. by measuring perceived social support), such as the Social Support Questionnaire and the Multidimensional Scale of Perceived Social Support, will be excluded.

Studies that use a measure that combines objective quantification of social isolation with the subjective measuring of perceived social support, such as the Duke Social Support Index 35‐Item Scale (George, Blazer, Hughes, & Fowler, 1989) and the Duke Social Support Index 10‐Item Scale (Wardian, Robbins, Wolfersteig, Johnson, & Dustman, 2013), will only be included if the results of the objective subscales or scale domains are reported separately.

This systematic review will be comprehensive regarding the timing of these measurements. In other words, we will include:

• Studies that have assessed an outcome once during the post‐intervention period (immediately after the intervention or in the longer term);
• Studies that have assessed the same outcome multiple times during the post‐intervention period (e.g. immediately after the intervention and 6 months later);
• Studies that have assessed the same outcome before the start of the intervention and post‐intervention.

Studies will not be excluded solely on the basis of reporting of outcome data. To this end, we will contact the authors to ascertain whether the data for our outcomes of interest are unavailable due to lack of measurement or lack of reporting.

#### 3.1.6 Secondary outcomes

Depressive symptom experiencing, life satisfaction and mental health outcomes will be considered as secondary outcomes.

### 3.2 Search methods for identification of studies

A comprehensive search for eligible published and unpublished studies and reports will be performed to reduce the risk of publication bias and identify the best available evidence. No date, location or language restrictions will be placed on the searches or included studies.

#### 3.2.1 Electronic searches

##### Electronic databases

The following databases will be searched from inception to present:

• The Cochrane Library (Cochrane Database of Systematic Reviews and Cochrane Central Register of Controlled Trials);
• MEDLINE (PubMed interface);
• Embase (Embase.com interface);
• PsycInfo and PsycArticles (psycnet.apa.org);
• ProQuest Sociology Database;
• Social Sciences Citation Index (Web of Science).

On the basis of previously published relevant papers and our selection criteria, a sensitive search strategy will be developed by JL and EDB, researcher and senior researcher at the Centre for Evidence‐Based Practice, where evidence‐based guidelines and systematic reviews are developed on a daily basis.

The strategy will be tailored to each specific database and will comprise both index terms (when relevant; e.g. MeSH terms, Emtree terms) and free text words (in title or abstract), with attention to possible synonyms, spelling variants, and correct use of truncation and proximity operators. Search filters will not be used, as they may prevent the retrieval of relevant papers.

De‐duplication of the references will be done using the EndNote reference management software (EndNote, 2013). All searches and search dates will be documented.

Below, the search strategy for MEDLINE (PubMed interface) is provided:

1. “Aged”[Mesh] OR elderly[TIAB] OR “Retirement”[Mesh] OR retire*[TIAB] OR pension*[TIAB] OR “old people”[TIAB] OR “older adults”[TIAB] OR “older people”[TIAB] OR ((resident[TIAB] OR residents[TIAB]) AND (“retirement home”[TIAB] OR “retirement homes”[TIAB] OR “nursing home”[TIAB] OR “nursing homes”[TIAB]))
2. "Volunteers"[Mesh] OR volunt*[TIAB] OR “friendly visitor”[TIAB] OR “friendly visitors”[TIAB] OR “friendly visiting”[TIAB] OR “friendly visit”[TIAB] OR “friendly visits”[TIAB] OR befriending[TIAB]
3. Visit*[TIAB]
4. “Social Isolation”[Mesh:NoExp] OR “Social isolation”[TIAB] OR “Loneliness”[Mesh] OR lonel*[TIAB] OR “Depression”[Mesh] OR depression[TIAB] OR depressive symptom*[TIAB] OR “Life satisfaction”[TIAB] OR “Mental health”[Mesh] OR “mental health”[TIAB]
5. 1‐4 AND

##### Grey literature sources and hand‐searching

We will consult the following sources of grey literature, and search the websites of organisations devoted to the specific topics of loneliness and ageing, to identify relevant unpublished studies and reports:

- Grey literature:
  ◦ Grey literature repositories:
    - Grey Literature Report (www.greylit.org);
    - OpenGrey (www.opengrey.eu);
    - ClinicalTrials.gov (clinicaltrials.gov);
    - International Clinical Trials Registry Platform of the World Health Organisation (ICTRP, apps.who.int/trialsearch/Default.aspx).
  ◦ Other sources of grey literature:
    - Google Scholar (scholar.google.be).
- Loneliness:
  ◦ Campaign to end loneliness in the UK (www.campaigntoendloneliness.org);
  ◦ Age UK (www.ageuk.org.uk/our‐impact/policy‐research/loneliness‐research‐and‐resources);
  ◦ No Isolation in Norway (www.noisolation.com/global/research/);
  ◦ Together against loneliness by Coalitie Erbij in The Netherlands (In Dutch: Eén tegen eenzaamheid; www.eentegeneenzaamheid.nl);
  ◦ Friends for Good in Australia (www.friendsforgood.org.au);
- Ageing:
  ◦ Age UK (www.ageuk.org.uk/our‐impact/policy‐research/publications/);
  ◦ Centre for Ageing Better (www.ageing‐better.org.uk/publications):
  ◦ International Longevity Centre UK (ILCUK, ilcuk.org.uk/reports/);
  ◦ WHO Ageing and life‐course Program (www.who.int/ageing/data‐research/en/);
  ◦ National Ageing Research Institute (NARI) in Victoria, Australia (www.nari.net.au/publications/overview‐about‐publications).

In compiling this list of organisations, the following criteria were applied:

- On their websites, the included organisations should explicitly state or show that they perform or bundle evaluations or reports on the effectiveness of (reading/ageing) interventions. In addition, these evaluations or reports should be readily available on their websites.
- University research groups were not included, as we expect them to publish their work in peer‐reviewed journals.

#### 3.2.2 Searching other resources

##### Other reviews

The reference lists of the above‐identified systematic reviews on the effectiveness of interventions aiming to reduce loneliness or social isolation will be scanned for relevant references.

##### Reference lists

The reference lists of included references will be searched. In addition, the ‘Related Articles' feature of the databases, if present, will be used.

##### Contacting experts

This review will be conducted in close collaboration with the Social Care Department of Belgian Red Cross. This Department runs a friendly visiting program, in which volunteers pay regular visits to older adults to tackle their feelings of loneliness and social isolation.

Furthermore, the review team will also receive content support from an external panel of social care experts (Vonk3 research centre of Thomas More University, Expertise centre Dementia Flanders, residential care centres, Public Centre for Social Welfare, Christian health insurance fund). These experts will be contacted to help identify other relevant studies.

### 3.3 Data collection and analysis

#### 3.3.1 Selection of studies

Study selection will be performed independently and in parallel by two evidence reviewers (JL and HS) in EndNote. In a first phase, titles and abstracts of the references identified by the search will be screened. Full texts of potentially relevant papers will be retrieved, and references that meet the selection criteria will be included for further analysis. Any relevant retraction statements and errata will be examined. In addition, relevant conference abstracts identified through the above‐mentioned searches will be included. Studies that meet the selection criteria and had the outcomes of interest measured, but do not report these outcome data, will be included and described in the Results section of the review.

Any discrepancies between the two reviewers will be resolved by consensus, and in case of disagreement a third reviewer will be involved (EDB). A PRISMA study selection flow chart will be provided (Moher, Liberati, Tetzlaff, Altman and The PRISMA Group, 2009) and a table of ‘Characteristics of excluded studies' will be presented in the final review.

#### 3.3.2 Data extraction and management

Data concerning the year in which the study was reported, the setting, the study design, and the basic characteristics of the study participants, interventions, and outcome measures will be independently extracted by the two reviewers. To ensure consistency in the data collection process, a standardised and piloted data collection form will be used (see Appendix).

By documenting all eligible available outcome measures in the ‘Characteristics of included studies' table, the two reviewers will be able to assess the potential for multiplicity of outcomes within the same study and handle them appropriately, following the guidance of the Cochrane Handbook for Systematic Reviews of Interventions (McKenzie et al., 2019).

If multiple methods are used to measure the same outcome within the same study, the reviewers will select the most relevant measure for analysis using the following decision rules:

- Outcomes measured via validated formal scales are more relevant than those measured using a single‐item question.
- Clinician‐rated outcome measures are more relevant than self‐reported measures.

If a single study has measured the same outcome at multiple time points, the reviewers will extract data from one short‐term time point (≤1 month after the intervention has ended), one intermediate‐term time point (>1 and ≤6 months after the intervention has ended) and one long‐term time point (>6 months after the intervention has ended).

If a single study only reports a composite measure of two or more of the outcomes of interest, the composite will be extracted and analysed.

If a study both contains data on overall scale findings, but also on the different dimensions addressed by the scale, only the overall scale results will be extracted.

During extraction, special attention will be paid to ensure that multiple reports of the same study are not treated as multiple studies. Should a study contain multiple intervention arms, the reviewers will only extract data on the intervention and control groups that are eligible for this review. Should a multi‐arm study report multiple relevant intervention arms, the findings from the different arms will be reported and analysed separately. Experimental and observational studies will be extracted and analysed separately.

For dichotomous outcomes, the number of events and the number of participants in each (intervention or control) group will be extracted. Odds ratios or risk ratios (both crude and adjusted ratios, if available) will be extracted, along with their 95% confidence intervals (CIs) and *p*‐values.

For continuous outcomes that can be assumed normally distributed, we will extract means, standard deviations (or information to estimate standard deviations), and the number of participants in each group. For skewed continuous data, medians, ranges, and *p*‐values of non‐parametric tests will be extracted.

In case of controlled before and after studies, mean or median change‐from‐baseline scores will be extracted, or computed by the reviewers if all necessary data are available. If change scores are not available or cannot be computed, post‐intervention values will be extracted by the reviewers.

Any discrepancies between the two reviewers will be resolved through discussion or consulting other review co‐authors.

#### 3.3.3 Assessment of risk of bias in included studies

Individual studies will be assessed for risk of bias, independently by the two reviewers. For randomised controlled trials, the Cochrane Risk of Bias tool will be used to identify the methodological quality and potential shortcomings therein (Higgins & Green, 2011). Study quality of non‐randomised experimental and observational studies will be assessed using the Risk of Bias In Non‐randomised Studies ‐ of Interventions (ROBINS‐I) tool (Sterne et al., 2016).

Next, the GRADE approach will be used to assess the overall certainty of the evidence included in this review, based on the limitations in study design, imprecision, inconsistency, indirectness, and publication bias (Atkins et al., 2004; Schünemann, Brozek, Guyatt, & Oxman, 2013). The certainty of the ‘body of evidence' will be assigned, ranging from high, moderate, low to very low.

#### 3.3.4 Measures of treatment effect

Continuous outcomes will be reported as mean differences (MD) with 95% CIs, whereas dichotomous outcomes will be reported as odds ratios (OR) or risk ratios (RR) with 95% CIs.

A ‘Summary of findings' table will be provided in the review, containing a summary of the results of all the included studies.

#### 3.3.5 Unit of analysis issues

Should we encounter a multi‐arm study, we will pay caution to ensure that the same group of participants is not included twice in a single meta‐analysis. In addition, paired data will be analysed appropriately.

#### 3.3.6 Dealing with missing data

In case of missing data, we will contact the authors at least twice to obtain these data, if correspondence details are available.

Where possible, we will calculate missing values (e.g. change scores, risk ratios, 95% CI and *p*‐values) from the available data, using the Review Manager 5 software (Higgins, Li, & Deeks, 2019; Review Manager, 2014). If insufficient data are available to calculate missing values, we will only analyse the available data and describe the results from the studies with missing data narratively.

In the final review, the issue of missing data and their potential impact on the findings will be discussed in the Discussion section.

#### 3.3.7 Assessment of heterogeneity

Forest plots will be inspected to visually investigate overlap in the confidence intervals for the results of the individual studies. The chi‐squared test will be performed and the *I* ^2^ statistic will be calculated to quantify inconsistency across studies. For the chi‐squared test, a *p*‐value of .10 will be used as a threshold for statistical significance. An *I* ^2^ threshold of 60% will be adopted. However, following the guidance of the Cochrane Handbook for Systematic Reviews of Intervention (Deeks, Higgins, & Altman, 2019), care will be taken in interpreting the results, should studies be few in number or have small sample sizes.

#### 3.3.8 Assessment of reporting biases

If 10 or more studies are identified, publication bias will be assessed through visual inspection of funnel plots. If the funnel plot shows asymmetry, a formal statistical Egger test will be performed. If there is evidence of funnel plot asymmetry from a test, we will attempt to distinguish the different possible reasons for this (non‐reporting biases, poor methodological quality leading to spuriously inflated effects in smaller studies, true heterogeneity, artefactual, chance) (Page, Higgins, & Sterne, 2019).

#### 3.3.9 Data synthesis

Experimental and observational studies will be analysed separately. Should cluster randomised controlled trials be included, they will be scrutinised and, if necessary, their analyses will be adjusted for clustering.

If two or more studies are identified that have investigated the effect of the same intervention on the same outcome, and data are sufficiently available, these data will be pooled and random‐effects meta‐analyses will be performed due to the expected between‐study variation, using the Review Manager 5 software. The Mantel‐Haenszel method and the Inverse‐Variance method will be used for dichotomous and continuous outcomes, respectively. Meta‐analysis results will be visually presented in forest plots. Change scores and post‐intervention values will be combined in the same meta‐analysis using the MD approach, in accordance with the guidance of the Cochrane Handbook for Systematic Reviews of Interventions (Deeks et al., 2019).

Should we encounter a combination of dichotomous and continuous data for the same outcome or predictor, we will first try to resolve this issue by collecting missing data from the study authors. If it remains impossible to summarise the results from all the relevant studies in a similar way, we will report and analyse the dichotomous and continuous data separately (Deeks et al., 2019).

In case a quantitative synthesis is not possible, study findings will be synthesised narratively, taking into account the overall certainty of the body of evidence.

#### 3.3.10 Subgroup analysis and investigation of heterogeneity

If substantial statistical heterogeneity is detected, heterogeneity may be explored by conducting subgroup analyses or (if at least 10 studies are included in the meta‐analysis) by conducting meta‐regression to guard against potential issues of confounding (Deeks et al., 2019). Heterogeneity may occur due to

1. Housing situation: In contrast to nursing home residents, who experience a certain degree of social interaction with other residents and care personnel on a daily basis, community‐dwelling older adults may live their lives with minimal social interaction. Therefore, it is conceivable that the effect of friendly visiting will be larger in community‐dwelling older adults compared to institutionalised older adults.
2. Activities engaged in during friendly visits: We hypothesise that friendly visiting that includes the use of interactive materials (e.g., playing checkers, dominoes, jigsaw puzzles) will have a more profound beneficial effect on loneliness and social isolation, compared to friendly visiting where the volunteer only engages in conversation and other types of social interaction (e.g., taking a walk) with the older adult.
3. Frequency and duration of visits: Friendly visiting programmes that invest in high‐frequency visiting and/or longer visits by a volunteer may have a more substantial impact on loneliness and social isolation, compared to low‐frequency and/or short‐duration friendly visiting.
4. Diversity at the level of gender, race/ethnicity, culture and geo‐political region: Friendly visiting programmes aimed at alleviating loneliness and social isolation may affect older adults differentially across different gender, race/ethnicity, culture and geo‐political region.

As direct analysis of more than two subgroups is not possible in the Review Manager 5 software, subgroups will be compared two by two, whether the outcome is continuous or dichotomous. *P*‐values will appropriately be adjusted for multiple testing.

Should post hoc subgroup analyses be conducted, we will clearly state in the review that these analyses are post hoc and exploratory in nature.

If a sufficient number of studies are identified, meta‐regression will be conducted using the R statistical software package, as this is not possible in the Review Manager 5 software.

### 3.4 Sensitivity analysis

Sensitivity analyses may be performed with respect to the quality of studies to test the robustness of the meta‐analysis by assessing whether results are not influenced by the inclusion or exclusion of low‐quality studies.

## CONTRIBUTIONS OF AUTHORS

J. L. drafted the protocol. All authors reviewed the draft and approved the final version.

## DECLARATIONS OF INTEREST

J. L., H. S., P. V. and E. D. B. are employees of the Belgian Red Cross and have no further interests to declare. One of the activities of the Belgian Red Cross is to run a friendly visiting program, in which volunteers pay regular visits to older adults to tackle their feelings of loneliness and social isolation.

## SOURCES OF SUPPORT

Internal sources

- Belgian Red Cross, Belgium

This systematic review is funded by the Foundation for Scientific Research of the Belgian Red Cross.

## EXTERNAL SOURCES

- No sources of support provided

## APPENDIX 1 DATA COLLECTION FORM

**Table:**

**Characteristics of included studies**				
Author, year, country	Study design	Population	Comparison	Remarks
**Synthesis of findings**				
Outcome	Comparison	Effect size	# studies, # participants	Reference
